# The Impact of the COVID-19 Pandemic on the Interest in Antidepressants: An Analysis of Worldwide Internet Searches With Google Trends Data

**DOI:** 10.7759/cureus.45558

**Published:** 2023-09-19

**Authors:** Ayşe Erdoğan Kaya, Nur Banu Oğur

**Affiliations:** 1 Psychiatry, Hitit University Çorum Erol Olçok Training and Research Hospital, Çorum, TUR; 2 Computer Engineering, Sakarya University, Sakarya, TUR

**Keywords:** depression, covid-19 pandemic, mental health, google trends, antidepressants

## Abstract

Objective: It is known that in the digital age we live in, people try to get information on many medical issues through Internet searches. Especially as a result of the COVID-19 pandemic triggering mental problems and health professionals' stay-at-home warnings, it has become difficult for individuals to receive psychiatric help, and this has encouraged accessing information about mental problems and their treatments through Internet searches. In this context, infodemiologic research, especially with Google Trends (GT; Google LLC, Mountain View, California, United States), has become very popular in recent years. In our study, it was aimed to examine the interest in frequently used antidepressants and the effect of the COVID-19 pandemic on Internet searches.

Methods: Search densities for five antidepressant drugs (sertraline, fluoxetine, citalopram, venlafaxine, duloxetine) that are frequently used around the world were examined on GT on 24/07/2023, and these searches were compared. Searches made within the last five years (24/07/2018-24/07/2023) were included in this study. Images were obtained using GT and Microsoft Excel 2019 (Microsoft Corporation, Redmond, Washington), and appropriate statistical analyses were performed with the SPSS Statistics version 22 (IBM Corp. Released 2013. IBM SPSS Statistics for Windows, Version 22.0. Armonk, NY: IBM Corp.).

Results: Sertraline was the most sought-after antidepressant before, during, and after the COVID-19 pandemic in the world. The searches related to sertraline increased gradually during the pandemic period, and this increase continued in the post-pandemic period. Other antidepressants whose search for it increased with the pandemic are fluoxetine, duloxetine, and venlafaxine. Searches for citalopram decreased during the pandemic process compared to the pre-pandemic period.

Conclusion: According to worldwide Internet searches, the prominence of some antidepressant group drugs during the pandemic period may be a reflection of the effects of the COVID-19 pandemic on mental health. Additionally, GT can provide psychiatrists with valuable insights into which depression medications are gaining popularity with the general public over time.

## Introduction

The widespread use of the Internet in society causes individuals to search public databases on subjects they are curious about or want to have information about [1]. Providing easy and fast access to leads in this way is reflected in searches made on medical issues as well as in every other subject [2]. Revealing the interest of individuals in society on any subject has gained popularity as digital field research in recent years [2]. In addition, the fact that the pandemic restricts real field studies and the inadequacy of epidemiological data can be considered as a situation that directs researchers to digital studies. Considering all these, Google Trends (GT; Google LLC, Mountain View, California, United States) also provides great opportunities for health research as it provides easily accessible and free data, and search activities can be followed in almost real-time. Infodemiological studies in the field of mental health are quite remarkable examples in this regard, and the increase in the number of these studies in recent years is striking [3,4].

Major depression, which is defined as a common mood disorder seen in approximately 15% of people and has the potential to lead to suicide, impairs functionality by reducing the person's interest in daily activities and is an important mental problem worldwide [5]. Both antidepressants and psychotherapeutic methods are used in the treatment. Antidepressant drugs show their effects as enzyme or receptor inhibitors and reuptake inhibitors [5]. Antidepressants that inhibit serotonin reuptake, a widely used group of drugs, are called selective serotonin reuptake inhibitors (SSRIs), and drugs such as sertraline, fluoxetine, paroxetine, fluvoxamine, and citalopram are in this group [5]. Another frequently used antidepressant group is serotonin and noradrenaline reuptake inhibitors (SNRIs), which provide antidepressant activity by inhibiting both serotonergic and noradrenergic reuptake. The drugs in the SNRI group are mainly venlafaxine, duloxetine, and milnacipran [5]. While the number of clinical studies on psychiatric diseases such as major depression is quite high, studies on the popularity of these diseases and their treatments in the digital field have started to gain popularity in recent years [2,6,7]. As with other health problems, individuals with depression also tend to research what they wonder about their illness and treatment through Internet searches. In particular, the fact that the COVID-19 pandemic triggered mental illnesses and individuals' difficulties in accessing health services may have caused an increase in Internet searches about mental health. There are many GT studies investigating the effect of the pandemic on mental health, but the number of studies on the frequency of searching for therapeutic techniques and pharmacological agents used to treat these mental problems is insufficient [8,9]. Our research aims to reveal the worldwide public interest in the drugs commonly used in the treatment of depression and to examine the effect of the pandemic on it.

## Materials and methods

Our research was designed as a cross-sectional and observational study based on GT data. Our study was conducted in accordance with the Principles of the Declaration of Helsinki. Ethics committee approval was not required as the data was obtained from the publicly available and free-of-charge website (https://trends.google.com/trends/).

GT data collection

GT is a free and publicly available database that provides a query index of terms that users type into the search engine using time intervals of hours, days, and years in a specific geographic region [2]. It provides the relative search volume (RSV) for each search term, allowing the search densities of the terms to be compared with each other over time [10]. Google search volume is normalized on a scale of 0 to 100 and is determined as RSV [10]. An RSV value of 100 represents the highest search volume over a period of time, while a value of 0 represents the lowest search volume. GT also provides an average weekly RSV value for that topic [10].

We separately obtained the Google search volume for antidepressant drug names on 25/07/2023. A worldwide search was made using the search terms "sertraline," "fluoxetine," "citalopram," "venlafaxine," and "duloxetine" in "all categories" and included data from the last five years. Five antidepressant drugs commonly used around the world were included in the analysis. The distribution over time of the resulting search volumes and the map of worldwide relevance for search terms were obtained from GT. The data covering the years 2020-2022 were accepted as the times when the effect of the COVID-19 pandemic was actively seen all over the world, and the change in search volumes in these years was examined. Since the first COVID-19 case worldwide was seen at the end of 2019, the period from 2018 to the beginning of 2020 was accepted as the pre-pandemic period, and the period from the beginning of 2023 was considered as the post-pandemic period. In each of the three periods, the search volumes for the search terms were compared over the mean weekly GT data. Search data for five commonly used antidepressant drugs were included in our study, as GT can compare up to five search terms at once.

Statistical analysis

Statistical analyses for the increase in antidepressant searches were performed with SPSS Statistics version 22 (IBM Corp. Released 2013. IBM SPSS Statistics for Windows, Version 22.0. Armonk, NY: IBM Corp.). The comparison of the mean weekly search volumes of the drugs included in our study was analyzed with appropriate statistical methods. For non-normally distributed continuous data, the Mann-Whitney U test was used for comparisons between two groups, and the Kruskal-Wallis Test was used for comparisons of more than two groups. The significance level was accepted as p<0.05. Other graphical analyses were obtained with Microsoft Excel 2019 (Microsoft Corporation, Redmond, Washington).

## Results

**Figure 1:**
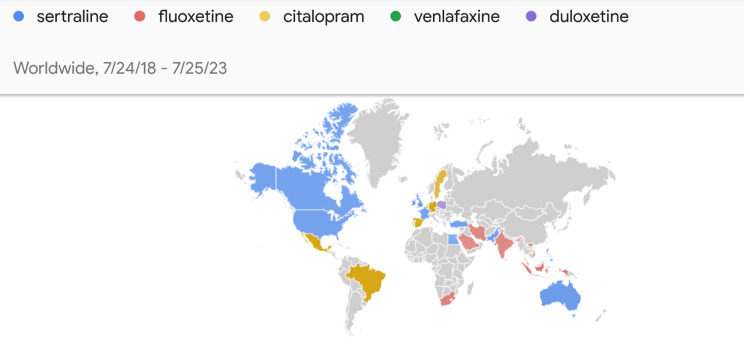
World map showing the intensity of antidepressant searches Derived from GT (https://trends.google.com/trends) (date of access 25/07/2023) Source: Reference [11]

**Figure 2:**
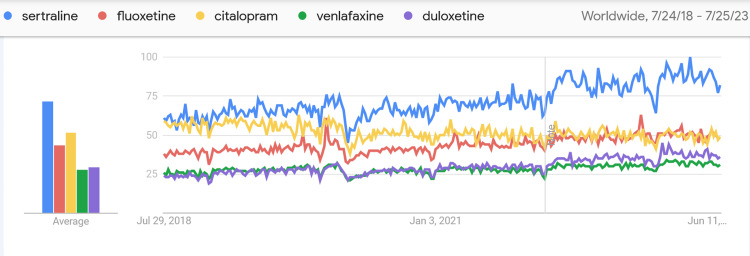
Graph of antidepressant search trends over time around the world Derived from GT (https://trends.google.com/trends) (date of access 25/07/2023) Source: Reference [11]

According to the data obtained from GT, the distribution of Internet searches for sertraline, fluoxetine, citalopram, venlafaxine, and duloxetine over time and the intensity by country are shown in Figure 1. The most sought-after antidepressant worldwide in the last five years is sertraline. According to the data obtained from GT, the graph of the change in search volumes over time is shown in Figure 2.

**Figure 3:**
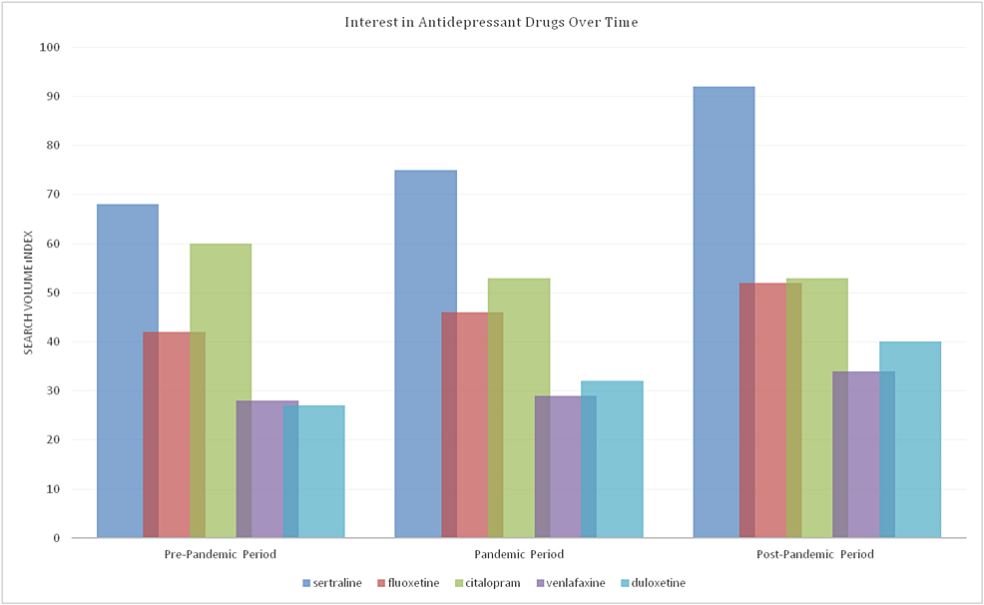
Interest in antidepressant drugs over time

The distribution of Internet searches for antidepressants during the COVID-19 pandemic process is seen in Figure 3, and it has been determined that the searches for sertraline and fluoxetine have increased during the pandemic and post-pandemic period. Again, there was an increase in searches for venlafaxine and duloxetine over time compared to the pre-pandemic period. The most frequently searched period for citalopram is the pre-pandemic period, and with the pandemic process, searches for citalopram have gradually decreased.

Considering the most frequently asked questions related to antidepressant drugs on GT, the most frequently asked question was what the side effects of the related drug were for all drugs.

As seen in Table 1, significant differences were found between each of the pre-pandemic, pandemic, and post-pandemic periods for sertraline, fluoxetine, venlafaxine, and duloxetine. For citalopram, while there was a significant difference between the pre-pandemic period and other periods, no difference was found between the pandemic and the post-pandemic period.

**Table 1:** Comparison of the average search frequency of antidepressant drugs on Google during the pre-pandemic, pandemic, and post-pandemic periods x̄: mean, SS: standard deviation * Kruskal-Wallis test (χ2), p<0.05 was significant

Antidepressant drug	Pre-pandemic (x̄ ± SS)	Pandemic (x̄ ± SS)	Post-pandemic (x̄ ± SS)	χ2	p
Sertraline	67.6 ± 4.4	75.3 ± 9.2	92.5 ± 4.1	102.731*	<0.001
Fluoxetine	42 ± 2.6	46.2 ± 5	52.5 ± 2.8	96.119*	<0.001
Citalopram	60.1 ± 3.8	52.7 ± 3.9	52.6 ± 2.2	114.975*	<0.001
Venlafaxine	23.4 ± 1.8	29.3 ± 2.6	33.6 ± 1.3	76.367*	<0.001
Duloxetine	26.8 ± 2.4	31.50 ± 4	39.2 ± 1.9	122.971*	<0.001

## Discussion

Our research, as an example of infodemiological research, constitutes an effective example of gaining rapid information about clinical situations and detecting psychiatric needs at an early stage, especially by examining Internet searches related to mental problems. The main purpose of our research was to investigate the intensity of antidepressant searches over the Internet in the last five years and the effect of the COVID-19 pandemic on this. Our main findings were that Google searches for antidepressants other than citalopram increased with the onset of the pandemic, and this increase continued after the pandemic. The most sought-after antidepressant drug was sertraline before, during, and after the pandemic. Citalopram, on the other hand, turned out to be an antidepressant drug whose frequency of searches decreased with the onset of the pandemic. In our study, the SSRI group of drugs was searched more than the SNRI group, and the results were consistent with studies on the frequency of clinical use of these drugs [12].

When the literature on this subject and previous studies are examined, no GT analysis studies for antidepressant searches on the Internet have been detected. However, there are many clinical studies and digital infodemiological studies focusing on the psychological effects of the COVID-19 pandemic [13-15]. Reasons such as the social isolation of the society within the scope of quarantine measures, the increase in concerns about the pandemic, and the primary mental effects of being infected with COVID-19 may have affected the mental health of individuals [16-18]. A study examining the effects of the pandemic on mental health in the United Kingdom in 2021 revealed that suicidal thoughts increased significantly, especially in young individuals [19]. Another study conducted in the USA found that symptoms of anxiety and depression increased with the pandemic [20].

The fact that sertraline has been known as a safe and frequently used drug for many years has been reflected in Google search volumes, and it has been determined that it is the antidepressant drug that attracts the most attention from society. Similarly, the fact that fluoxetine is a safe and frequently preferred drug may have contributed to its increased popularity and Internet searches during the pandemic period. The fact that duloxetine and venlafaxine are also sought more in the post-pandemic period compared to the pandemic period may be a reflection of the need for stronger drugs with the increase in mental health problems after the pandemic. Studies on this subject, in line with our findings, drew attention to the long-term mental effects of the pandemic and suggested increasing the psychological resilience of individuals against possible new pandemics [21].

Another important finding of our study is that the increase in the search for antidepressant drugs other than citalopram continues in the post-pandemic period. This may be related to the persistence of its psychological effects despite the end of the pandemic. As a matter of fact, when clinical studies in the post-pandemic period were examined, it was understood that anxiety and circadian rhythm disorders continued, that is, the psychological effects of the pandemic were long-lasting [22]. No concrete (drug interaction, efficacy, etc.) reason could be identified to explain the decreased search frequency for citalopram. However, factors such as the advertising strategies of pharmaceutical companies and prescribing preferences of physicians may be the underlying causes.

Besides the antidepressant names in GT, the most searched phrases were what the side effects were for all drugs. This may be due to curiosity or lack of knowledge about the side effects of people seeking treatment as well, or individuals may not be able to obtain satisfactory information from healthcare professionals about the drugs they continue to use or are prescribed to them. This may be due to the weak patient-physician relationship from time to time, the limitation of examination times, the restriction of face-to-face patient-physician meetings within the scope of quarantine, and the treatment-related concerns of the patients. Clinical studies measuring patients' perspectives, attitudes, and knowledge levels about psychiatric treatments have also addressed the need for more satisfactory information on these issues [7,23].

Our research seems remarkable in that it is infodemiological research that reveals the digital reflection of the mental problems triggered by the COVID-19 pandemic, especially the anxiety disorders and depressive disorders in which antidepressants are used in the treatment and the need for treatment. On the other hand, this study has some limitations. GT only shows the interest of people with Internet access and people who use the Google search engine. It may also have limited validity in regions with low Internet use or freedom of expression. Because of all this, although infodemiological studies are not as valuable as real epidemiological studies, they seem to give an early and quick idea about how some medical issues are on the agenda.

## Conclusions

Our GT study including pre-pandemic, pandemic, and post-pandemic processes revealed the change in the search volume of antidepressants and the impact of the COVID-19 pandemic on it. This study can be considered as the triggering effect of the pandemic on mental illnesses or as a reflection of the increase in seeking treatment in the digital world. The use of the Internet as an important resource on mental health makes research on this subject valuable and provides an easily accessible way for mental health professionals to access infodemiological information.
